# Identification of carcinogens by a selected panel of DNA damage response associated genes 

**DOI:** 10.17179/excli2015-766

**Published:** 2015-12-22

**Authors:** Regina Stöber

**Affiliations:** 1 Leibniz Research Centre for Working Environment and Human Factors at TU Dortmund (IfADo), Ardeystrasse 67, 44139 Dortmund, Germany

## ⁯⁯⁯⁯

Recently, Bettina Maria Fischer and colleagues of the Institute of Toxicology in Karlsruhe have published data on a gene expression based predictive test system for chemical carcinogens (Fischer et al., 2015[8]). The authors used bronchial epithelial cell lines and analyzed the expression of 95 genes by a high-throughput RT-qPCR system. The analyzed genes cover the biological motifs DNA damage response, genomic stability, cell cycle control, apoptosis and mitotic signaling (Fischer et al., 2015[8]). In a case study using the carcinogenic compound cadmium, the authors demonstrate that genes involved in the DNA damage response were up-, while DNA repair genes were down-regulated, thereby giving a clear-cut positive result in the low micromolar concentration range.

Identification of chemical carcinogens represents a cutting-edge topic in toxicology (Liu et al., 2015[15]; Ustündag et al., 2014[22]; Bustaffa et al., 2014[4]; Bach et al., 2014[1]; Seiler et al., 2001[21]; Westphal et al., 2000[25]; Venkov et al., 2000[23]). Since it is not possible to test all chemicals in long-term carcinogenicity rodent studies, fast but nevertheless accurate predictive tests are urgently needed (Zhang et al., 2015[27]; Ireno et al., 2014[12]; Kumar and Dhawan, 2013[14]; Mohiuddin et al., 2014[17]; Bertini et al., 2000[3]; Ostby et al., 1997[19]). Gene array studies have been frequently applied to characterize the impact of chemicals on genome-wide expression patterns in an unbiased manner (Cunningham, 2001[6]; Ellinger-Ziegelbauer et al., 2008[7]; Nie et al., 2006[18]; Krug et al., 2013[13]; Rempel et al., 2015[20]; Lohr et al., 2015[16]; Campos et al., 2014[5]; Balmer et al., 2014[2]). However, it has also been suggested that smaller subsets of genes may be sufficient for characterizing expression responses to chemicals due to the redundancy of highly correlated gene clusters (Waisberg et al., 2003[24]; Grinberg et al., 2014[11]; Godoy et al., 2015[10], 2013[9]). This offers the advantage to use quantitative RT-PCR techniques instead of more cost intensive array based or sequencing approaches (Zellmer et al., 2010[26]). In this context the test system presented by Fischer et al. (2015[8]) represents an attractive approach. Of course validation studies will have to be performed, including sufficiently high numbers of positive and negative control compounds also in comparison to the already established bacterial and mammalian mutagenicity tests.
